# Ammonium Hydroxide Pretreatment of Sugarcane Bagasse and Corn Cob: Saccharification Enhancement and Ethanol Production by C6/C5 Cofermentations

**DOI:** 10.1021/acsomega.6c01259

**Published:** 2026-10-05

**Authors:** Andressa Moreira do Nascimento, Gisele de Fátima Leite Souza, Daniel Pasquini, Milla Alves Baffi

**Affiliations:** † Chemical Institute (IQ-UFU), 425927 Federal University of Uberlândia, 38400-902 Uberlândia, Minas Gerais, Brazil; ‡ Agricultural Sciences Institute (ICIAG-UFU), 28119 Federal University of Uberlândia, 38405-320 Uberlândia, Minas Gerais, Brazil

## Abstract

The pretreatment of lignocellulosic biomass is a key step in second-generation (2G) ethanol production. In this study, sugarcane bagasse (SCB) and corn cob (CC) were pretreated with 28–30% ammonium hydroxide (1:10 w/v ratio, 30 min, 180 °C) to promote lignin removal and enhance enzymatic hydrolysis. Delignification levels reached 65.5 and 73.5% in pretreated (PT) SCB and CC, (PTSCB and PTCC, respectively), with preservation of the polysaccharide fraction. The biomasses were submitted to enzymatic saccharification using Cellic CTec3 (12.5 FPU/g cellulose) at solid concentrations of 10–30%. Fed-batch hydrolysis with 30% solids released the highest total reducing sugars (TRS), achieving 65.15 g/L in PTSCB and 70.18 g/L in PTCC, with increases of 238 and 137% in comparison to the batch at 10% of substrate. The hydrolysates presented low levels of fermentative inhibitors and high concentrations of glucose (43.75 and 41.63 g/L) and xylose (18.12 and 18.75 g/L) in PTSCB and PTCC, respectively. The fermentation of PTCC hydrolysate with the nonconventional *Spathaspora passalidarum* Y7204 monoculture reached the ethanol production (26.34 g/L, *Y*_p/s_ = 0.44, and *Q*_p_ = 0.73 g/L·h), while the commercial *Saccharomyces cerevisiae* Y904 yielded 19.18 g/L (*Y*_p/s_ = 0.47; *Q*_p_ = 0.80 g/L·h). The cocultures of both yeasts generated around 20 g/L of ethanol; however, metabolic interactions and catabolic repression may have occurred. These findings evidenced the NH_4_OH pretreatment as a promising strategy for enhancing saccharification. In addition, the study highlighted the positive role of *S. passalidarum* in the hexose (C6) and pentose (C5) cofermentation of PTCC hydrolysate.

## Graphical abstract

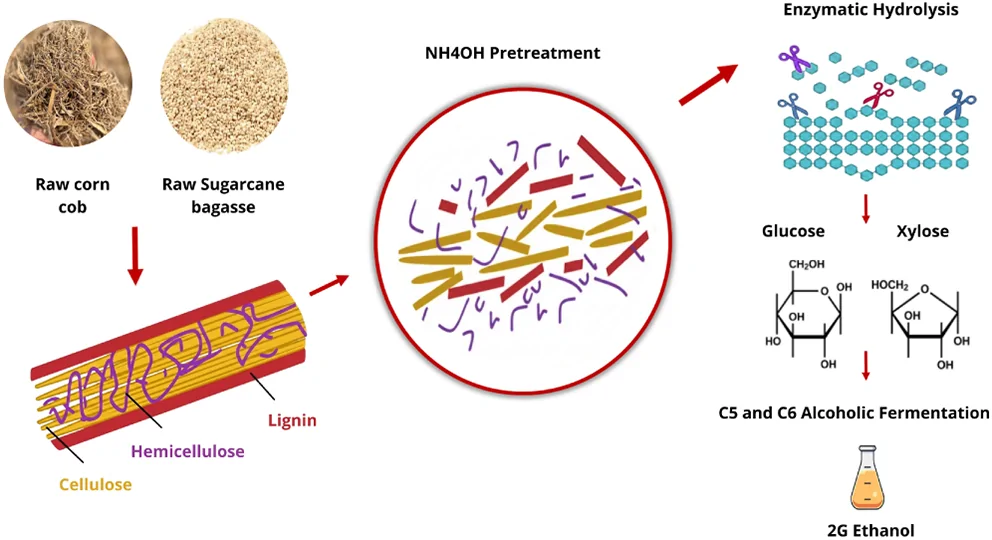

## 1 Introduction

Energy dependence is a growing global challenge, intensified by rapid population growth and economic expansion.^1^ The predominant use of fossil fuels exposes countries to substantial risks, such as the volatility of international markets, geopolitical tensions, and the depletion of natural resources. In this scenario, the search for renewable sources, such as biofuels, with less environmental impact becomes essential for the diversification of the energy matrix. Among biofuels, second-generation ethanol (2G), produced from agro-industrial byproducts rich in lignocellulosic biomass, such as sugarcane bagasse and corn cobs, represents a promising alternative.^2^ However, the complex and recalcitrant nature of these materials, associated with cellulose crystallinity, restricts their efficient conversion into fermentable sugars.^3^ Thus, biomass pretreatment is a crucial step to increase the surface area, reduce crystallinity, and make cellulose more accessible for enzymatic hydrolysis.^4^

Among the available pretreatments, the alkaline method has demonstrated effectiveness in the removal of lignin and acetyl groups from hemicellulose, facilitating cellulose accessibility. Among them, the use of ammonium hydroxide (NH_4_OH) has demonstrated advantages due to porosity improvement and minimal inhibitor formation. Unlike many conventional pretreatments, ammonia selectively disrupts lignin–carbohydrate linkages while preserving most cellulose and hemicellulose, resulting in high polysaccharide recovery and improved enzymatic digestibility. Ammonia also induces fiber swelling, making them more accessible to enzymes, thereby optimizing the reaction and increasing sugar yields.^5^ Furthermore, ammonia-based pretreatments are noncorrosive, nontoxic, and pollution-free, and present harmless operation and reusability.^6^ The mild operating conditions also minimize the formation of inhibitory compounds, enhancing downstream hydrolysis and fermentation efficiencies. Another advantage of ammonia is its volatility, which facilitates its recovery by depressurization and phase change after the pretreatment, reducing chemical consumption, waste generation, and operating costs. Furthermore, it can be used as a nitrogen source for yeasts during fermentation and the nitrogen-containing liquors generated may be valorized as fertilizers or soil amendments, promoting nutrient recycling and supporting a circular bioeconomy. Moreover, ammoniation of the methoxyl groups of lignin avoids the nonproductive adsorption of enzymes to lignin.^7,8^ These features make ammonia-based pretreatment an attractive approach for next-generation biorefineries focused on efficient biomass conversion and sustainability.

Another important factor is the saccharification step performed with commercial enzymatic cocktails, containing a variety of cellulases and hemicellulases, which act synergistically to degrade cellulose and hemicellulose, promoting the efficient depolymerization of these substrates.^9^ In this stage, the use of high-solids loading in fed-batch mode has demonstrated high effectiveness in sugar release.^10,11^ In this process, the challenges associated with medium viscosity, reduced mass transfer, and enzyme inhibition by pretreatment products are overwhelmed by the gradual addition of biomass to the reaction.^11^

In the last step, the sugars released in the hydrolysates can be fermented and converted into ethanol. In alcoholic fermentation, the yeast *Saccharomyces cerevisiae* has been predominantly employed due to its rapid growth, high efficiency in hexose (C6) fermentation, and tolerance to environmental stresses.^12^ However, this species is not capable of fermenting the pentoses (C5) derived from the hemicellulosic fraction, reducing ethanol yields. As an alternative, the use of nonconventional yeasts, such as *Spathaspora passalidarum*, has gained attention due to its ability to coferment glucose and xylose, increasing 2G ethanol productivity.^13,14^ Moreover, the whole sugar utilization from lignocellulosic feedstock can be attained by microbial cocultures, in which *S. cerevisiae* successfully ferments glucose, and the residual xylose can be metabolized by C5-fermenting yeasts.^15^ Thus, the synergistic interaction between these species can provide improvements in the fermentative process.

In this context, the present study evaluated the effect of NH_4_OH pretreatment on the enzymatic fed-batch saccharification of sugarcane bagasse (SCB) and corn cob (CC). Then, the hydrolysates were fermented, using *S. cerevisiae* and *S. passalidarum*, in monocultures or in mixed and sequential cofermentations, aiming to enhance 2G ethanol production.

## 2 Research Methodology

### 2.1 Alkaline Pretreatment

Sugarcane bagasse (SCB) was provided by Usina Vale do Tijuco (Uberaba, MG, Brazil), and corn cob (CC) was provided by JC Rações e Insumos Siderúrgicos Ltda. (Uberlândia, MG, Brazil). The biomasses were milled and sieved (10 mesh) to obtain particles around 2 mm. The alkaline pretreatment was carried out in a Parr bench-scale reactor (Parr Instrument Company, model 4848), equipped with a heating system and temperature regulator, with 28–30% of NH_4_OH, at a 1:10 (w/v), for 30 min at 180 °C.^8^ Then, the residual biomasses were filtered, washed with approximately 500 mL of deionized water, and frozen at −20 °C. The assays were performed in triplicate, and average values were reported.

### 2.2 Characterization of Biomass

#### 2.2.1 Quantification of Total Klason Lignin, Cellulose, and Hemicellulose

Raw and pretreated SCB and CC were characterized for total Klason lignin (acid-soluble lignin and acid-insoluble lignin), cellulose, hemicellulose, moisture, extractives, and ash.^16^ For the lignin determination, the samples were hydrolyzed with 72% sulfuric acid (H_2_SO_4_) at room temperature for 1 h. The acid was diluted to 3%, and the mixture was heated to approximately 100 °C under reflux for 1 h (TAPPI T222 om-88). The residual material was cooled and filtered through a glass filter (number 3). The solids were dried to constant weight at 105 °C. The cellulose and hemicellulose contents were determined using a CR7A SHIMADZU chromatograph with an R10-6A refractive index detector and an Aminex HPX-87H column (300 × 7.8 mm, Bio Rad). The eluent used was H_2_SO_4_ 0.005 mol/L with a flow rate of 0.6 mL/min (NREL LAP-002). The delignification rate and the yield of pretreated samples were determined according to eqs 1 and 2:

1 $$\%\;\mathrm{delignification}=\frac{{\mathrm{LK}}_{\mathrm{biomass}}-\left(\frac{{\mathrm{LK}}_{\mathrm{pulp}}\times R}{100}\right)}{{\mathrm{LK}}_{\mathrm{biomass}}}\times 100\%$$

where LK_biomass_ corresponds to the Klason lignin of the original biomass, LK_pulp_ refers to the Klason lignin of the biomass after pretreatment, and *R* corresponds to the mass yield of the pretreatment:

2 $$R=\frac{m_{\mathrm{biomass,initial}}-m_{\mathrm{biomass,after}}}{m_{\mathrm{biomass,initial}}}\times 100\%$$

where *m*_biomass,initial_ is the initial biomass mass and *m*_biomass,after_ is the biomass mass after pretreatment.

The moisture content was determined according to TAPPI T264 cm-97. Ash content was measured by the percentage difference between the initial dry fiber weight and the weight after calcination at 800 °C for 4 h (TAPPI T211 om-02). The determination of extractive content soluble in cyclohexane/ethanol or water was performed according to TAPPI T204 cm-97.

#### 2.2.2 X-ray Diffraction

X-ray diffraction (XRD) was executed using a Shimadzu XRD6000 instrument (Shimadzu, Japan), working at 40 kV with a current of 3 mA and Cu Kα radiation (1.5406 Å). The 2θ scan data were detected from 10° to 35° at a scan rate of 1.0°/min. The crystallinity index (CI), which indicates the relative degree of crystallinity, was calculated using eq 3:^17^

3 $$\mathrm{CI}=\left(\frac{I_{200}-I_{\mathrm{am}}}{I_{200}}\right)\times 100\%$$

where *I*_200_ refers to the maximum intensity, in arbitrary units, at the crystalline peak at around 2θ = 22° and *I*_am_ is the intensity of the amorphous area at 2θ = 18°.

#### 2.2.3 Scanning Electron Microscopy (SEM)

The raw and pretreated SCB and CC were morphologically examined with a CARL ZEISS MOD EVO MA10 microscope using an acceleration voltage of 5.0 kV. Before analysis, each sample was coated with an ultrathin layer of gold through a sputter-coating system. The images were obtained at magnifications of 100×, 500×, and 1000×.^10^

#### 2.2.4 Fourier Transform Infrared Spectroscopy (FTIR)

The infrared absorbance spectra of raw and pretreated SCB and CC were examined using a Shimadzu IRPrestige-21 spectrometer. The samples were pelletized at a concentration of 1:100 (w/w) of potassium bromide (KBr) for succeeding evaluations at the regions 4500 to 350 cm^–1^, with resolution of 4 cm^–1^ and 32 scans.^10^

### 2.3 Conventional Enzymatic Hydrolysis

The saccharifications of raw and pretreated SCB and CC were conducted in 50 mL Erlenmeyer flasks containing sodium citrate buffer (0.005 mol/L, pH 4.8), with a liquid-to-solid ratio of 10 mL/g (10% of the substrate) and 12.5 FPU (filter paper units) of the commercial solution Cellic CTec3 (Novozymes) per gram of cellulose.^10^ The assays were performed at 150 rpm and 50 °C for 72 h. The reactions were performed in triplicate, and average values were reported. The hydrolysates containing the released sugars were centrifuged at 8760*g* for 10 min and membrane-filtered (0.20 μm, Chromafil Xtra CA-20/25). The total reducing sugars (TRS) were quantified by the 3,5-dinitrosalicylic acid (DNS) method, using 100 μL of hydrolysate and 100 μL of DNS solution, which were heated at 100 °C for 10 min in a water bath. Then, 800 μL of distilled water was added to the reaction mixture, and the absorbance was read using a UV–Vis spectrophotometer (Metash, model UV-5100, Shanghai, China) at 540 nm.^10^

### 2.4 Fed-Batch Saccharification

The fed-batch hydrolysis was carried out under the same previous experimental conditions but with additions of 5% substrate every 12 h (at 12, 24, 36, and 48 h) until a total of 30% solids was reached.^13^ After 72 h, the liquid fractions containing the released sugars were filtered and centrifuged. The TRS concentration was determined by the DNS method.^11^

The concentrations of glucose, xylose, arabinose, cellobiose, acetic acid, formic acid, furfural (FF), and 5-hydroxymethylfurfural (HMF) released after the fed-batch saccharifications were determined by high-performance liquid chromatography (HPLC). The samples were diluted with the mobile phase (aqueous phosphoric acid solution at 0.1% (v/v), filtered (0.20 μm, Chromafil Xtra CA-20/25), and introduced into the chromatographic system (Shimadzu LC-20A Prominence). The system was equipped with a Supelcogel C-610H column maintained at 32 °C, using isocratic elution at a flow rate of 0.5 mL/min. Ultraviolet detectors (operating at 210 nm) and refractive index detectors were used. The analysis was based on glucose, xylose, organic acids, furfural (FF), and hydroxymethylfurfural (HMF) standards.^11^

The enzymatic bioconversions (EC) were calculated according to eqs 4 and 5:

4 $${\mathrm{EC}}_{\mathrm{glucose}}=\left(\frac{m_{\mathrm{glucose}}f_{\mathrm{hg}}}{m_{\mathrm{sample}}y_{\mathrm{ic}}}\right)\times 100\%$$

5 $${\mathrm{EC}}_{\mathrm{xylose}}=\left(\frac{m_{\mathrm{xylose}}f_{\mathrm{hx}}}{m_{\mathrm{sample}}y_{\mathrm{ix}}}\right)\times 100\%$$

where *m*_glucose_ and *m*_xylose_ (g) are the weights of glucose and xylose released from the hydrolysis of glucan (cellulose) and xylan (hemicelluloses), respectively; *m*_sample_ (g) is the sample weight used in the assay; *f*_hg_ = 0.9 and *f*_hx_ = 0.88 are the conversion factors, which take into account that one water molecule was added during cellobiose and xylobiose breakage; and *y*_ic_ and *y*_ix_ (%) are the contents of glucan and xylan in the substrate before hydrolysis, respectively.

### 2.5 Alcoholic Fermentation

Four fermentation assays of pretreated SCB and CC hydrolysates were investigated: (1) monoculture of *Saccharomyces cerevisiae* Y-904 for 72 h (commercial Fleischmann’s baker’s yeast); (2) monoculture of *Spathaspora passalidarum* UFMG-CM-Y7204 for 96 h; (3) simultaneous coculture of *S. cerevisiae* Y-904 and *S. passalidarum* Y7204 for 96 h; and (4) sequential coculture, beginning with *S. cerevisiae* Y-904 at time zero and with the addition of *S. passalidarum* Y7204 after 24 h, with a total duration of 120 h. The wild-type *S. passalidarum* Y7204 strain was originally isolated from the gut of a beetle and kindly donated by the Federal University of Minas Gerais (UFMG). This isolate was preserved at −80 °C in the Microorganism and Cell Collection of UFMG (a public microbial collection). These yeast strains were individually precultivated in a rotary shaker at 30 °C and 180 rpm for 24 h, in 5 mL of YPD medium composed of (w/v) 1% yeast extract, 2% peptone, and 2% glucose. The preinocula were obtained from cells recovered by centrifugation at 2600*g* for 15 min, washed with sterile saline solution (0.85%), and resuspended in the fermentation media at an initial cell concentration of 0.5 g/L.^18^

For the fermentation, the hydrolysates (10 mL) from the fed-batch hydrolysis of the pretreated biomass were used. PTSCB and PTCC hydrolysates were autoclaved (at 0.5 atm) for 15 min, supplemented with yeast extract (3%), and added in 50 mL Erlenmeyer flasks. Then, the flasks were sealed with hydrophobic cotton plugs and sterilized gauze and incubated at 30 °C and 180 rpm for 72, 96, and 120 h, according to the respective fermentation strategy, in triplicate.^18^ The fermentative assays were monitored by the extraction of aliquots at 0, 24, 48, 72, 96, and 120 h, according to the total duration of each assay. Afterward, the samples were centrifuged for 15 min at 2600*g* and filtered (0.20 μm, Chromafil Xtra CA-20/25), and analyzed by HPLC to determine the concentrations of sugars, ethanol, organic acids, FF, and HMF.^19^

### 2.6 Fermentation Parameters

The fermentation parameters were calculated according to Campos et al.^20^ The ethanol yield (*Y*_p/s_) was calculated using eq 6:

6 $$Y_{p/s}=\frac{\left(P_{f}-P_{i}\right)}{\left(S_{i}-S_{f}\right)}$$

where *P*_f_ is the final mass of ethanol (g), *P*_i_ is the initial mass of ethanol (g), *S*_i_ is the initial mass of sugar (g), and *S*_f_ is the final mass of sugar (g).

The ethanol volumetric productivity (*Q*_p_, g/L·h) was estimated using eq 7:

7 $$Q_{p}=\frac{\left(P_{f}-P_{i}\right)}{t}$$

where *P*_f_ is the final ethanol concentration (g/L), *P*_i_ is the initial ethanol concentration (g/L), and *t* is the fermentation period (h).

The fermentation efficiency (η) was estimated using eq 8, in which the experimental *Y*_p/s_ (g/g) was divided by the theoretical *Y*_p/s_ (0.51 g/g), and the result was multiplied by 100%.

8 $$\eta =\frac{Y_{p/s\left(\mathrm{experimental}\right)}}{Y_{p/s\left(\mathrm{theoretical}\right)}}\times 100\%$$

## 3 Results and Discussion

### 3.1 Biomass Characteristics

#### 3.1.1 Chemical Composition

The chemical compositions of raw and pretreated biomasses were analyzed (Table 1). Raw SCB showed 43.11% cellulose, 23.30% hemicellulose, and 26.56% lignin. For raw CC, 31.76% of cellulose, 37.27% of hemicellulose, and 18.01% of lignin were observed, with varying levels of ash, extractives, and moisture (Table 1). These results are consistent with previous studies, which have reported similar compositions for sugarcane bagasse^21,22^ and corn cobs.^10,11^ The minor variations can be attributed to some factors, such as origin, age, climatic conditions, and harvesting/storage methods.^23^

**Table 1:** Chemical Composition (%) of Raw Sugarcane Bagasse (RSCB), Raw Corn Cob (RCC), NH_4_OH Pretreated Sugarcane Bagasse (PTSCB), and NH_4_OH Pretreated Corn Cob (PTCC)

	composition (%)			
	RSCB	PTSCB	RCC	PTCC
cellulose	43.11 ± 0.13	70.80 ± 0.81	31.76 ± 0.76	69.32 ± 0.71
hemicellulose	23.30 ± 0.71	21.57 ± 0.12	37.27 ± 0.81	24.14 ± 0.39
Klason total lignin	26.56 ± 0.48	18.85 ± 0.18	18.01 ± 0.44	9.12 ± 0.95
ash	0.60 ± 0.03	0.05 ± 0.01	0.55 ± 0.04	0.04 ± 0.03
extractive content	5.46 ± 0.09	–	5.87 ± 0.06	–
moisture	8.26 ± 0.46	7.74 ± 0.20	8.50 ± 0.43	7.83 ± 0.25
compositional sum	107.29 ± 0.31	119.01 ± 0.26	101.96 ± 0.42	110.45 ± 0.46
delignification	–	65.50 ± 0.01	–	73.50 ± 0.63

The NH_4_OH-pretreated sugarcane bagasse (PTSCB) exhibited 70.80% of cellulose, 21.57% of hemicellulose, and 18.85% of lignin, with a notable delignification rate of 65.50% and a yield of 49.09% (Table 1). Previous studies investigating sugarcane bagasse pretreatment with NH_4_OH have reported lignin removals between 35.96 and 77.46%, with the highest solubilization occurring around 180 °C.^23,24^ In the pretreated corn cob (PTCC), the composition was 69.32% cellulose, 24.14% hemicellulose and 9.12% lignin, resulting in a delignification rate of 73.5% and a yield of 54.69% (Table 1). These data corroborate the high efficiency of NH_4_OH in lignin removal from corn residues, with proportional increases in the cellulosic fraction.^25^ Although variations in the final composition can occur due to different pretreatment conditions (NH_4_OH concentration, solids loading, temperature, and reaction time), NH_4_OH is recognized for its effectiveness in the selective removal of lignin and hemicellulose, preserving the cellulose.^26^ The high delignification is attributed to the saponification of ester bonds between lignin and polysaccharides and the formation of ammonium and hydroxyl ions that break these ester bonds within the lignin–carbohydrate structure, which increase the biomass surface area and porosity, facilitating the enzymatic attack on the polysaccharide matrix in the subsequent saccharification step.^10,26^ In this sense, ammonia-based pretreatments preferentially reduce lignin in comparison to acid and hydrothermal pretreatments.^8^ Based on these data, NH_4_OH pretreatment positively contributed to the biomass disruption of SCB and CC.

#### 3.1.2 X-ray Diffractometry

XRD is a widely used analytical technique to identify and quantify crystalline structures and evaluate material properties.^27^ The diffractograms of raw SCB and CC (Figure 1) revealed peaks around 18° (amorphous halo) and 22.5° (crystalline peak), which are characteristic of cellulose type I, confirming the presence of this polymer, with ordered and partially crystalline arrangements.^27^ After the ammonia pretreatment, the crystallinity index (CI) increased from 63.27% in RSCB to 69.84% in PTSCB and from 55.34% in RCC to 63.17% in PTCC, which is consistent with previous studies that have also reported increases in crystallinity in alkaline-pretreated sugarcane and corn residues.^10,28^

**Figure 1:**
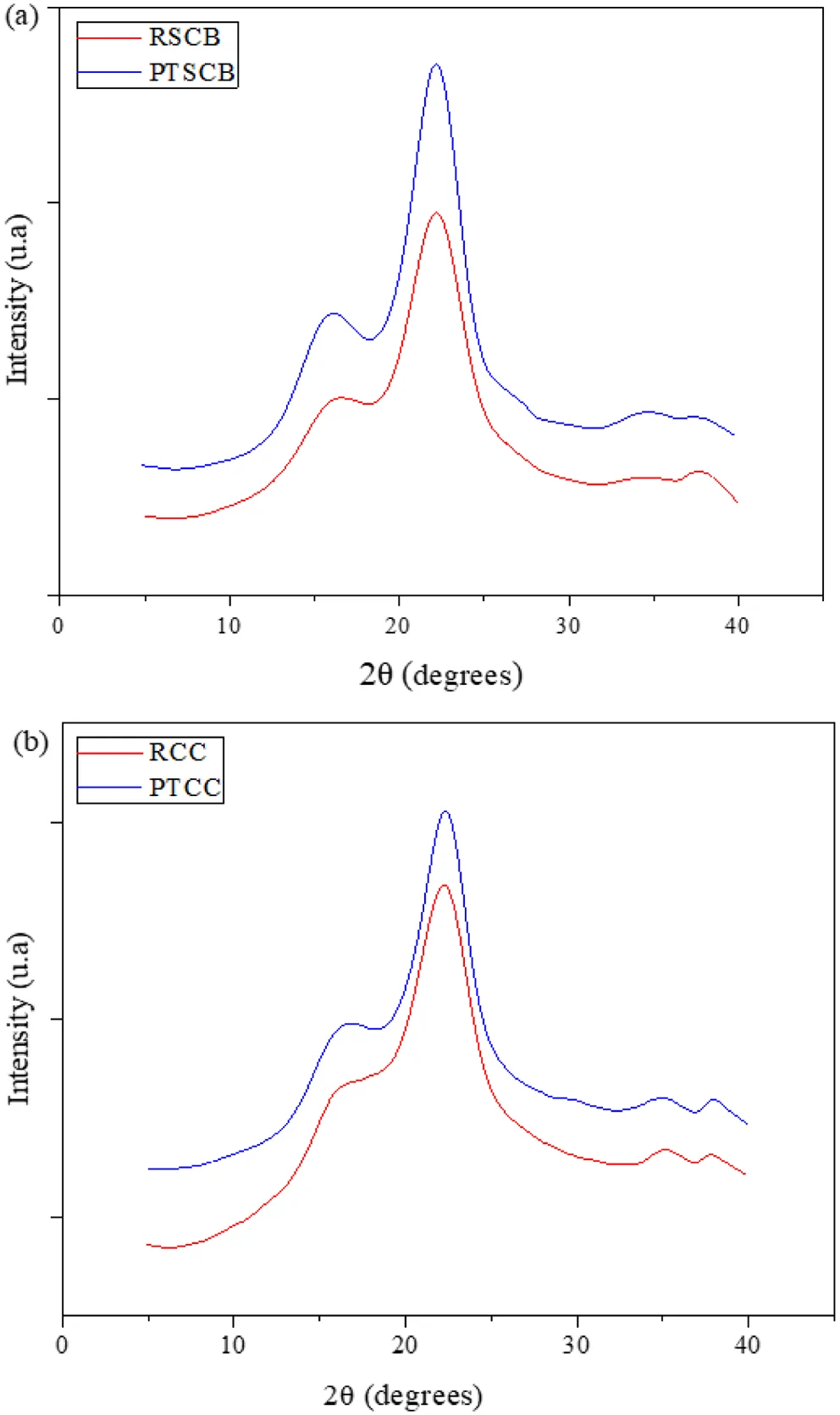
X-ray diffraction patterns of the raw and NH_4_OH-pretreated biomasses: (a) RSCB and PTSCB; (b) RCC and PTCC.

The NH_4_OH pretreatment influences cellulose crystallization, primarily by removing the amorphous fractions of lignin and hemicellulose, with temperatures above 130 °C promoting an elevated degree of crystallization.^29^ Thus, the observed crystallinity intensification in both PTSCB and PTCC is primarily attributed to the removal of these amorphous fractions. In addition, elevated crystallinity could also be associated with greater organization and compaction of cellulose chains.^29^ Furthermore, although it commonly indicates the relative proportion of crystalline and amorphous regions, the crystallinity index can include other components beyond the cellulosic fraction.^30^

#### 3.1.3 Fourier Transform Infrared Spectroscopy

FTIR was used to investigate changes in the chemical structure and functional groups of both raw and NH_4_OH-pretreated SCB and CC. In the raw materials, the spectra revealed absorption bands characteristic of lignocellulosic biomass. A broad band was identified between 3600 and 3200 cm^–1^, attributed to the hydrogen-bond (O–H) vibrations of cellulose hydroxyl groups (Figure 2). Bands in the 3000–2700 cm^–1^ region were also observed, corresponding to carbon–hydrogen (C–H) bond stretching in aliphatic chains. Additionally, the presence of aromatic groups from lignin and phenolic compounds was indicated by bands between 1700 and 1500 cm^–1^. In the 1300–800 cm^–1^ range, bands associated with hemicellulose functional groups were identified, while peaks between 930 and 875 cm^–1^ were related to the glycosidic bond stretching of cellulose and hemicellulose.^31^

**Figure 2:**
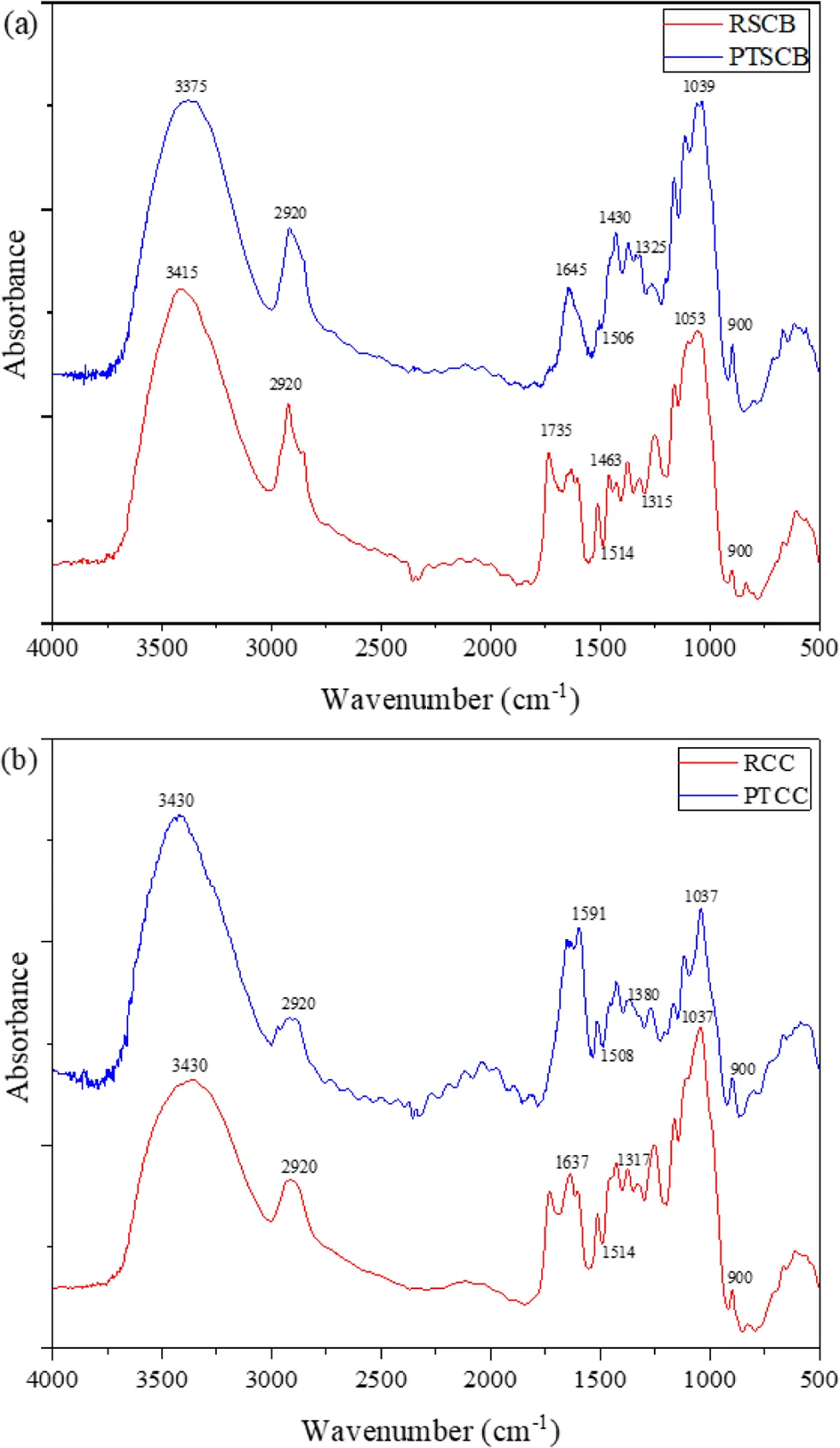
FTIR spectra of the raw and NH_4_OH-pretreated biomasses: (a) RSCB and PTSCB; (b) RCC and PTCC.

After the pretreatment, FTIR analysis indicated important changes in the structure and composition of PTSCB and PTCC. The absence of peaks in the 1700–1500 cm^–1^ range, along with the decline of existing bands, suggested lignin removal (Figure 2). A reduction in the peaks associated with hemicellulose was also observed, confirming the cleavage of its linkages. The cellulose structure was preserved, as evidenced by the maintenance of peaks between 1300 and 800 cm^–1^. Similar results were reported by Kumar et al.,^1^ who pretreated sugarcane bagasse with NaOH and observed the disappearance of peaks in the 1700–1500 cm^–1^ and 1300–800 cm^–1^ ranges, indicating the breakage of bonds within the lignin molecule. As well, the intensity of the bands at 930–875 cm^–1^ increased, which are associated with glycosidic bonds, indicating greater exposure of functional groups and an increase in the cellulosic fraction due to structural changes promoted by the alkaline pretreatment.^31^ Furthermore, the intensification in the 3000–2700 cm^–1^ range was attributed to the removal of complex compounds and aliphatic bonds that had masked the functional groups in the raw biomasses.^32^ Li et al.^33^ also demonstrated the partial removal of lignin and hemicellulose by NH_4_OH pretreatment, suggesting a reduction in the recalcitrance of the biomasses.

#### 3.1.4 Scanning Electron Microscopy (SEM)

The micrographs revealed marked changes on the surfaces of the pretreated biomasses compared with the raw samples (Figure 3). In SCB and CC, a compact and rigid morphology was observed, indicative of structural integrity. This rigidity is attributed to lignin, which functions as a protective layer for the fibrous matrix and is further reinforced by the presence of fatty acids and waxes.^34^

**Figure 3:**
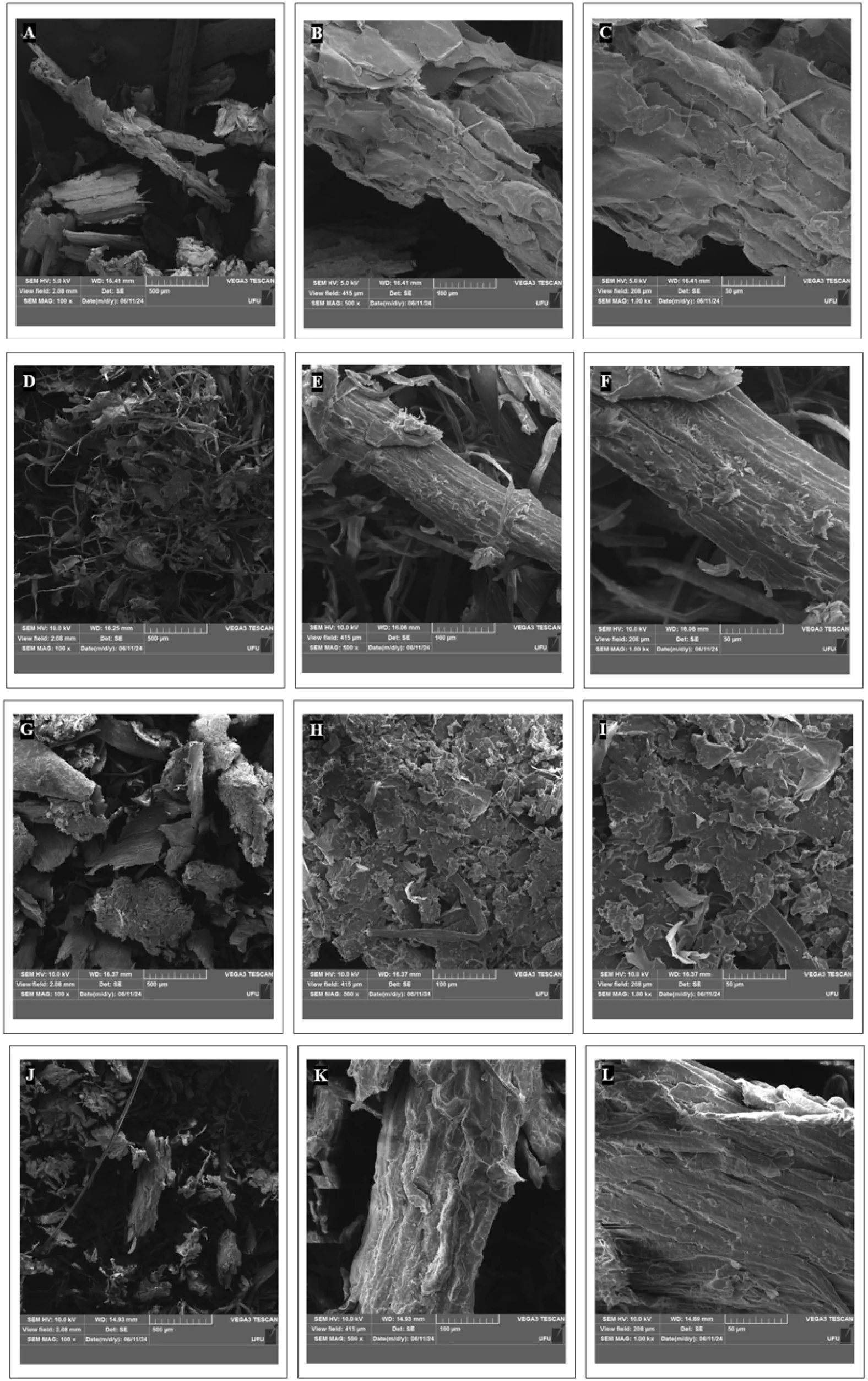
Electron micrographs of biomass surfaces: (A–C) RSCB, (D–F) PTSCB, (G–I) RCC, and (J–L) PTCC. Magnifications: 100× (A, D, G, J), 500× (B, E, H, K), and 1000× (C, F, I, L).

In the pretreated samples, the formation of pores and an increased surface area were evident and attributed to delignification. In addition, the PTSCB and PTCC displayed a smoother and more fibrous architecture, resulting from cellulose exposure. This increased porosity is likely derived from the partial removal of hemicellulose and lignin, which rendered additional fibers available for enzymatic digestion.^11^ Comparable morphological changes have been reported for rice straw, sugarcane bagasse, and corn stover pretreated with NaOH and NH_4_OH, highlighting the effectiveness of alkaline methods in disrupting the internal structure of lignocellulosic biomass.^34^ Therefore, the ammonia pretreatment markedly altered the biomass architecture, enhancing fiber flexibility and increasing the exposure of cellulose microfibrils for the subsequent saccharification stage.^35^

### 3.2 Conventional and Fed-Batch Hydrolysis

In the conventional enzymatic hydrolysis with 10% solids, the raw SCB and CC liberated 10.34 and 12.77 g/L of TRS, respectively. In PTSCB and PTCC hydrolysates, the TRS release reached 19.29 and 29.58 g/L, representing increases of 86.55 and 131.63%, respectively (Figure 4), thus demonstrating the positive effect of the NH_4_OH pretreatment on the hydrolytic efficiency, mainly due to the high delignification rate, especially in PTCC.^36^ Previous studies have demonstrated the positive role of alkaline pretreatments in saccharification. For example, Ascencio et al.^37^ reported 32.82 g/L of TRS in hydrolysates of NaOH-pretreated SCB using 15 FPU of Cellic CTec2/g of cellulose, with an increase of 68.78% compared with the raw biomass. Tsai et al.^38^ performed saccharification of Napier grass subjected to H_2_SO_4_ and NaOH pretreatments using 12 FPU of Cellic CTec2/g of cellulose and observed an increase of 75.4% in the glucose yield, indicating enhanced performance for the alkali-treated biomass due to the selective removal of lignin and hemicellulose.

**Figure 4:**
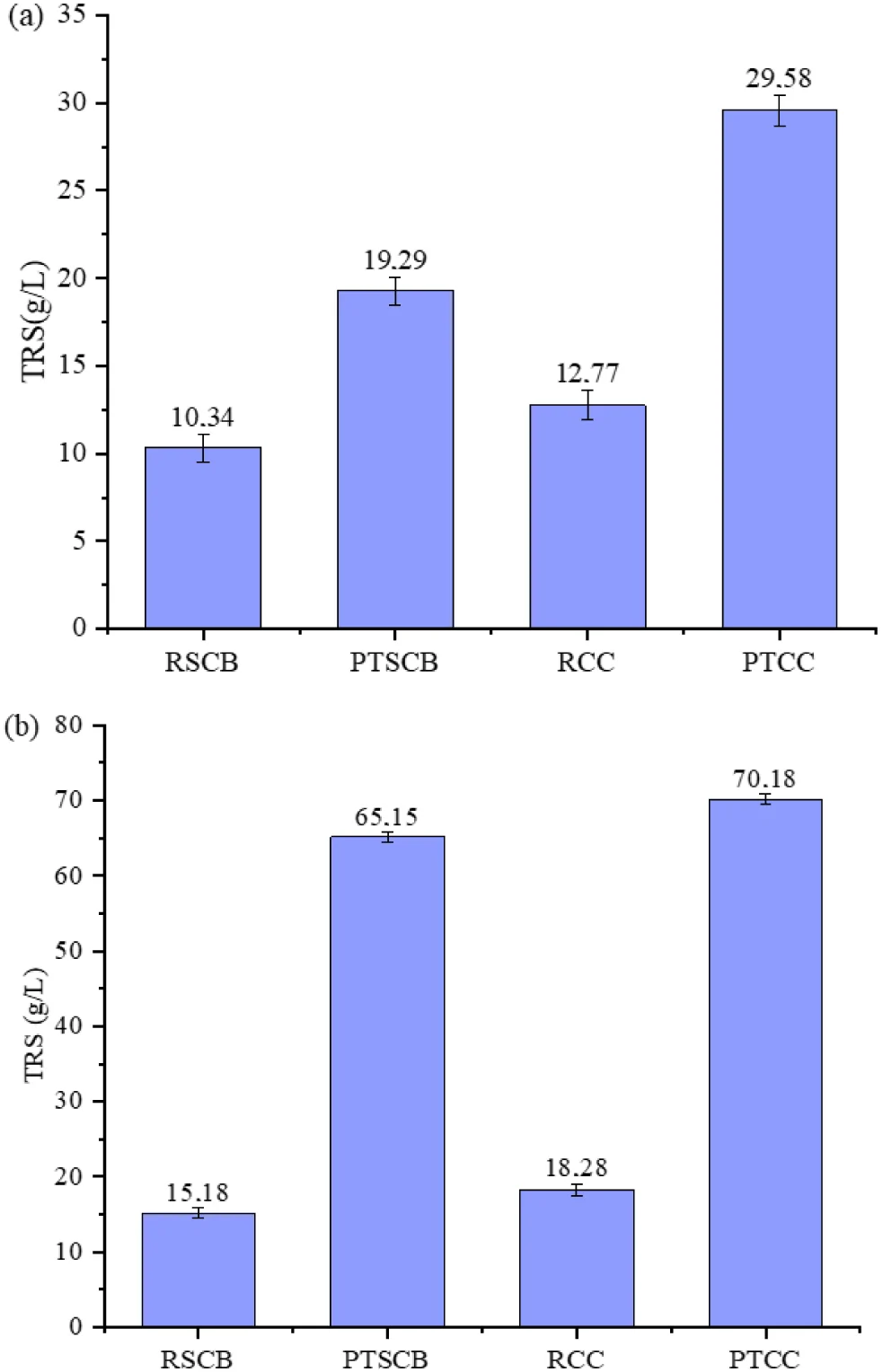
Total reducing sugars (TRS) content of the raw and NH_4_OH-pretreated biomasses after enzymatic hydrolysis with 10% solids (a) and in fed-batch mode with 30% solids (b).

Another factor that influences the yield of fermentable sugars is the biomass loading. In this regard, saccharifications at high solids percentages have been conducted to improve the hydrolytic process.^11^ However, elevated substrate concentrations can impair mixing, compromising mass and heat transfer and, consequently, the enzymatic efficiency.^39^ As an alternative, fed-batch saccharification with 30% of solids was carried out in the present study, resulting in 65.15 g/L of TRS in PTSCB and 70.18 g/L in PTCC (Figure 4). This TRS release represents increases of 238 (PTSCB) and 137% (PTCC) in comparison to conventional hydrolysis. These results are greater than those obtained by Jiménez et al.^24^ and Velmurugan et al.,^39^ who performed hydrolyses in fed-batch mode of NH_4_OH pretreated sugarcane straw and corn cob and observed 62.19 and 57.80 g/L of TRS, respectively. The data also indicate that PTCC showed improved hydrolytic efficiency than PTSCB, possibly due to its notable delignification rate (Table 1). The effectiveness of fed-batch saccharifications was also observed by Fornazier et al.,^11^ who carried out the hydrolysis of NaOH-pretreated corn cob, starting with 10% of solids loading, followed by gradual additions of 5% every 12 h, reaching 67.90 g/L of TRS with 30% substrate.

Furthermore, high concentrations of fermentable sugars, especially glucose and xylose, were observed in the fed-batch hydrolysates with 30% solids (Table 2). These contents were similar in the pretreated hydrolysates (43.75 and 41.63 g/L of glucose and 18.12 and 18.75 g/L of xylose in PTSCB and PTCC, respectively), indicating enhanced depolymerization of cellulose and hemicellulose. This increased hydrolytic efficiency may be due to the partial removal of lignin, which could have facilitated the enzymatic attack and contributed to the increase in the release of these monosaccharides.^39^

**Table 2:** Concentrations of Sugars, Organic Acids, and Inhibitors in Fed-Batch Hydrolysates of NH_4_OH-Pretreated SCB and CC^a^

	concentration (g/L)	
	PTSCB	PTCC
cellobiose	1.50 ± 0.44	0.27 ± 0.03
glucose	43.75 ± 1.40	41.63 ± 0.84
xylose	18.12 ± 1.16	18.75 ± 0.24
arabinose	0.97 ± 0.04	0.51 ± 0.06
formic acid	0.04 ± 0.02	ND
acetic acid	0.07 ± 0.,05	ND
5-hydroxymethylfurfural	0.02 ± 0.01	0.03 ± 0.01
furfural	0.04 ± 0.02	0.03 ± 0.02
EC_glucose_ ^b^	55.61	54.04
EC_xylose_ ^b^	73.92	68.35

a ND: Not detected.

b EC_glucose_ and EC_xylose_ (%) were calculated according to eqs 3 and 4 (Section 2.4).

On the other hand, pretreatments can generate undesirable byproducts, such as formic acid, acetic acid, FF, and HMF. These compounds are toxic not only for the enzymes during the hydrolytic process but also for the yeast strains involved in alcoholic fermentation.^4^ The concentrations of these inhibitors depend on some factors, such as biomass composition, type of pretreatment, pH, temperature, and pressure.^23^ These compounds can originate from hemicellulose degradation, which releases xylose, mannose, acetic acid, galactose, and glucose. Under high temperatures and pressures, xylose can be converted to FF. Similarly, HMF is derived from hexoses. The decomposition of FF and HMF leads to the formation of formic acid, while levulinic acid results from the transformation of HMF. Phenolic compounds derive from the partial decomposition of lignin but can also be produced during biomass degradation.^40^ At high concentrations (above 3.0 g/L), acetic and formic acids can exert inhibitory effects on the hydrolytic efficiency and are highly toxic to microbial growth. Moreover, FF and HMF, at concentrations of 2.0 and 4.0 g/L, respectively, reduce the efficiency of enzymatic hydrolysis and hinder the growth and metabolic activities of microorganisms.^40^ However, in this study, low concentrations of these compounds were detected in the hydrolysates, demonstrating the contribution of the NH_4_OH pretreatment in reducing byproduct formation (Table 2), which also minimizes sugar degradation. Pretreatment conditions can significantly influence the formation of inhibitory compounds. However, alkaline pretreatment is characterized by lower inhibitor formation, positively impacting the hydrolysis efficiency, in contrast to acid treatments, wherein a high formation of these compounds is observed.^41^ Therefore, the NH_4_OH pretreatment contributed to the formation of PTSCB and PTCC hydrolysates with low concentrations of these inhibitors, while high concentrations of fermentable sugars were achieved.

### 3.3 Alcoholic Fermentation

The PTSCB and PTCC hydrolysates were subjected to alcoholic fermentation using different culture strategies with *S. cerevisiae* Y904 and *S. passalidarum* Y7204 yeasts. Fermentation 1, carried out with *S. cerevisiae* monoculture, demonstrated total consumption of glucose, reaching ethanol concentrations of 21.39 and 19.18 g/L in PTSCB and PTCC, respectively, after 24 h of cultivation, with elevated yields, productivities, and fermentative efficiencies (Table 3). Comparable values were obtained by Wang et al.,^42^ who obtained 22.37 g/L of ethanol after fermentation was carried out with *S. cerevisiae* of NaOH-pretreated SCB hydrolysate. Fornazier et al.^10^ also reported nearby values (16.11 g/L; 0.67 g/g), using the same Y904 yeast isolate in the fermentation of NaOH-pretreated CC hydrolysate, containing 10% biomass. On the other hand, Fornazier et al.^11^ achieved 25.81 g/L of ethanol after the fermentation of NaOH-pretreated CC fed-batch hydrolysate, containing 35% of biomass. However, the fermentative parameters were similar among these studies (η, 88.88%). These variations may be due to the different pretreatments and hydrolysis conditions carried out in these studies. After 48 h, the ethanol concentration was reduced, which may be associated with osmotic stress, due to the high sugar concentrations in the hydrolysates or with inhibition of microbial metabolism by ethanol, as reported by Sica et al.^43^ Therefore, the obtained ethanol in Fermentation 1 indicated that, although the glucose conversion was complete, variables such as ethanol inhibition or cellular stress may have influenced its performance.^14,15^

**Table 3:** Ethanol Production from PTSCB and PTCC Hydrolysates by *S. cerevisiae* Y904 and *S. passalidarum* Y7204

fermentation	hydrolysate	glucose consumption (%)	xylose consumption (%)	sugar consumption (%)	ethanol (g/L)	*Y*_p/s_ (g/g)	η (%)	*Q*_p_ (g/L/h)	time (h)
1 (Y904)	PTSCB	100	–	–	21.39 ± 0.60	0.49 ± 0.01	96	0.89 ± 0.03	24
	PTCC	84	–	–	19.18 ± 1.63	0.47 ± 0.07	93	0.80 ± 0.07	24
2 (Y7204)	PTSCB	100	86	95	19.75 ± 0.99	0.31 ± 0.01	60	0.41 ± 0.03	48
	PTCC	100	82	91	26.34 ± 0.59	0.44 ± 0.01	87	0.73 ± 0.02	48
3 (Y904/Y7204)	PTSCB	100	76	91	15.25 ± 0.86	0.24 ± 0.01	48	0.64 ± 0.04	24
	PTCC	90	10	65	14.95 ± 0.91	0.38 ± 0.02	74	0.62 ± 0.04	24
4 (Y904/7204-24 h)	PTSCB	100	20	71	11.02 ± 0.03	0.24 ± 0.00	46	0.47 ± 0.00	24
	PTCC	94	42	78	16.60 ± 0.65	0.35 ± 0.02	69	0.35 ± 0.01	48

In Fermentation 2, *S. passalidarum* Y7204 monoculture cofermented glucose and xylose, resulting in the highest ethanol concentrations of 26.34 g/L (PTCC hydrolysate), with a yield of 0.44 g/g and a productivity of 0.73 g/L/h. These values surpassed those obtained with PTSCB hydrolysate (19.75 g/L; 0.31 g/g; 0.41 g/L/h), reflecting efficient sugar conversion due to the elevated delignification rate and biomass depolymerization in PTCC (Tables 1 and 2; Figure 4). This ethanol production is in agreement with previous studies carried out with *S. passalidarum*. For example, Nakanishi et al.^44^ reported 23.3 g/L of ethanol by *S. passalidarum* Y-27907 cultured in alkali-pretreated PTSCB hydrolysate, with similar yield and productivity (0.45 g/g; 0.81 g/L/h). Campos et al.,^20^ using synthetic media, also confirmed the fermentative capacity of this species, although with lower yields than the present study (0.28 g/g). These comparisons evidence the greater effectiveness of the Y7204 strain in fermenting the NH_4_OH-pretreated PTCC hydrolysate, probably due to the low concentrations of inhibitory compounds. However, despite its capacity to ferment xylose, the use of *S. passalidarum* in 2G ethanol production faces challenges regarding its metabolic role in lignocellulose-derived hydrolysates. In our results, xylose consumption was initiated just after glucose depletion (Figure 5), indicating that C5 metabolization in *S. passalidarum* was inhibited by C6. Previous studies have described that this inhibition occurs at the initial stage of glucose uptake, in the first steps of glycolysis.^44^ This phenomenon, known as carbon catabolic repression, leads to the prioritization of more energetically favorable sugars (C6) over the secondary ones (C5), extending the fermentation time and reducing the ethanol yield.^45^ This inhibitory effect of glucose on xylose is dose-dependent, indicating that high levels of hexose generated from enzymatic hydrolysis can influence pentose metabolism.^46^ This condition justifies the ethanol peak at 48 h of fermentation, followed by a decrease in its production after 72 h (Figure 5).

**Figure 5:**
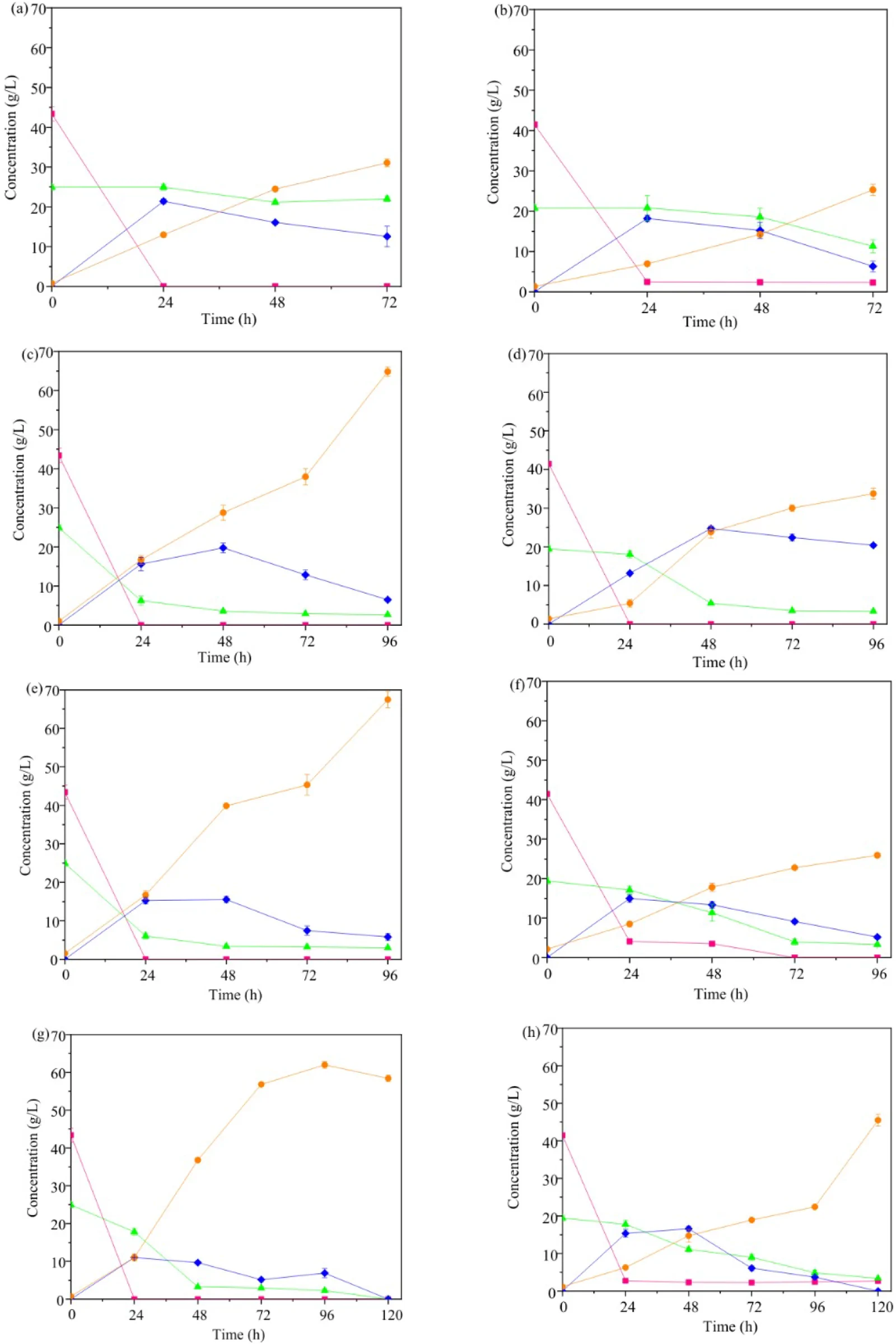
Alcoholic fermentation of PTSCB and PTCC hydrolysates. (a, b) Fermentation 1 with *S. cerevisiae* Y904 monoculture. (c, d) Fermentation 2 with *S. passalidarum* Y7204 monoculture. (e, f) Fermentation 3 with Y904 and Y7204 simultaneous coculture. (g, h) Fermentation 4 with Y904 and Y7204 sequential co-culture. Glucose (pink ■), Xylose (green ▲), Ethanol (violet ◆), and Biomass (yellow ●).

Regarding Fermentation 3, involving the simultaneous coculture of conventional *S. cerevisiae* Y904 and *S. passalidarum* Y7204, it resulted in 15.25 g/L of ethanol (PTSCB), with yields ranging from 0.24 to 0.38 g/g and efficiencies of up to 74%, which are consistent with Rech et al.^47^ and Rodrigues et al.,^48^ who found effectiveness between 55 and 95% in cofermentations using different yeast species combinations. Cocultures can allow each microorganism to metabolize its respective substrate and result in increased yields.^49^ However, in Fermentation 3, it is likely that the presence of glucose inhibited xylose metabolism by *S. passalidarum*, also due to catabolite repression.^20,45^ In this context, a competitive inhibition mechanism of glucose may be present, due to its higher affinity for the transporter, with xylose metabolization occurring only after C6 depletion.^45^ Recent evidence has indicated that high concentrations of hexoses can significantly hinder xylose consumption by *S. passalidarum*, with the inhibition occurring at the sugar uptake level, prior to glycolysis. This inhibition can involve glucose transport and cytosolic phosphorylation, leading to a signal transduction cascade that impairs C5 transport and delays its metabolism.^43^ A similar phenomenon was also reported by Wu et al.,^50^ who observed competition between *S. cerevisiae* and *S. stipitis* during the initial stages, with late adaptation of the second strain to xylose, which could have also occurred in the present study. This regulatory mechanism likely contributed to the slower or incomplete xylose consumption observed in mixed-sugar fermentations, despite the intrinsic capability of this yeast to metabolize pentoses. Moreover, although coculture strategies can broaden substrate utilization, the presence of elevated glucose concentrations probably intensified the competition between the yeast species and reinforced catabolite repression, delaying xylose assimilation by *S. passalidarum*. Similar interactions have been reported in mixed cultures, where glucose-preferring yeasts dominate the early stages of fermentation, negatively affecting pentose metabolism by nonconventional yeasts.^51^

In Fermentation 4, which was sequentially conducted with conventional *S. cerevisiae* and nonconventional *S. passalidarum* inoculated after 24 h, the lowest ethanol concentrations were obtained in comparison to the earlier conditions (11.02 g/L in PTSCB and 16.60 g/L in PTCC), with yields of 0.24 g/g and 0.35 g/g, respectively. Although xylose consumption has been observed, the ethanol already present in the medium may have inhibited the metabolism of the second strain.^14^ Okuofu et al.^52^ obtained similar results (9.45 g/L ethanol from cowpea waste) using sequential fermentation, emphasizing that while this approach can be effective, its success depends on the ethanol tolerance of the yeasts involved in the process. In addition, the main limitations of sequential fermentations include a slight reduction in ethanol productivity and incomplete xylose conversion when present in mixtures containing high sugar concentrations.^53^

Considering the fermentations carried out with *S. passalidarum* (Fermentations 2, 3, and 4), a noteworthy increase in xylose consumption was observed after 24 and 48 h, always after glucose depletion, as also evidenced in previous studies in which this yeast has exhibited high sensitivity to ethanol, with growth inhibition at concentrations above 20 g/L, representing a bottleneck for 2G ethanol expansion.^43,53^ Moreover, the results confirmed that *S. passalidarum*, both isolated and in coculture with *S. cerevisiae*, prioritizes glucose as the main carbon source, with xylose uptake occurring only after C6 depletion. However, further studies are needed to fully explore the regulatory role of hexoses in pentose metabolism in sequential mixed inocula. In this sense, the development of genetically modified strains with increased ethanol tolerance can be an important strategy in order to maximize the industrial use of nonconventional yeasts.^45^ In addition, despite the low inhibitor formation in the hydrolysates of PTCC and PTSCB, the inhibition of this yeast species by compounds originating from lignocellulosic pretreatments, such as acetic acid, FF, and HMF, even in very low concentrations, has also been reported and could have affected the fermentative efficiency.^43^ Thus, detoxification methods for inhibitor removal in lignocellulosic hydrolysates should be investigated in order to improve its tolerance and fermentation effectiveness.

The biomass analysis during the fermentations revealed increased cell growth after 48 h in all fermentations (Figure 5). This positive correlation between biomass increase and ethanol production reinforces the role of yeast metabolic activity up to the point of inhibition by ethanol.^14^ In the sequential coculture using PTSCB (Fermentation 4), an average reduction in cell density from 61.97 to 58.41% was observed, indicating that ethanol concentration may negatively affect cell growth. This effect can be associated with changes in cell wall permeability, resulting in irregularities in signaling functions and damage to the cell cycle.^54^ In addition, the inoculation of non-*Saccharomyces* strains may also be affected by the inoculation of *S. cerevisiae*, highlighting the complexity of microbial interactions in these fermentative systems. The results also showed that in the later periods of Fermentations 2, 3 and 4, microbial growth continued, indicating that pentose metabolism may have favored biomass production, instead of ethanol.^43^ Another factor to be considered is the aeration of the culture medium, which may influence the fermentative metabolism of the yeasts, directing it toward biomass production.^55^ This aspect can explain the ethanol peak at 48 h, in Fermentations 2, 3, and 4, followed by a gradual decline in its concentration, accompanied by xylose consumption, suggesting that xylose could be consumed more slowly and primarily used for cellular maintenance. In addition, another hypothesis is that *S. passalidarum*, under stress, may have consumed ethanol as an alternative carbon source, justifying the decrease in ethanol concentration at the end of the fermentations, even with high biomass concentrations.^56^ Therefore, adaptive evolution strategies and optimization of some parameters, such as temperature, inoculum size, and aeration, could be useful to mitigate catabolite repression, ethanol inhibition, and other stress factors.^57^

## 4 Conclusions

The combined strategy of ammonium hydroxide pretreatment, fed-batch saccharification, and the cofermentation of hexoses and pentoses using conventional and wild yeasts was compared between sugarcane bagasse and corncob for 2G ethanol production . The NH_4_OH pretreatment successfully achieved noteworthy delignification rates in PTSCB and PTCC, leading to elevated TRS releases after enzymatic hydrolysis, with low formation of inhibitory compounds, especially in PTCC. The fermentation of PTCC hydrolysate with *S. passalidarum* Y7204 reached the highest ethanol production (26.34 g/L ethanol and η = 87%). Cocultivation assays also showed high productivity, although the phenomenon of catabolite repression could have occurred. To the best of our knowledge, this is the first study which successfully demonstrated ethanol production by a wild *S. passalidarum* monoculture or in coculture with *S. cerevisiae*, in NH_4_OH-pretreated corn cob hydrolysates. Literature reporting this integrated strategy is scarce, with most of the studies chiefly describing other kinds of pretreatment, other lignocellulosic biomasses and genetic modified yeasts for this purpose. In addition, this study also compared the fermentative efficiency of *S. cerevisiae* and *S. passalidarum*, and the use of both yeasts in the same hydrolysate, expanding knowledge of the nonconventional yeasts’ potential. Further research will be needed to fully understand some factors, such as substrate competition and metabolic interactions, and extend the fermentative effectiveness for future industrial application.

## Data Availability

The authors declare that the data supporting the findings of this study are presented within the paper. Raw data will be available upon request.
